# Morphogenesis of honeybee hypopharyngeal gland during pupal development

**DOI:** 10.1186/s12983-017-0207-z

**Published:** 2017-04-20

**Authors:** Sascha Peter Klose, Daniel Rolke, Otto Baumann

**Affiliations:** 1 Institute of Biochemistry and Biology, Department of Animal Physiology, University of Potsdam, Karl-Liebknecht-Str. 24/25, 14476 Potsdam, Germany; 2 Present Address: Institute of Biology, Department of Molecular Parasitology, Humboldt University, Philippstrasse 13, 10115 Berlin, Germany

**Keywords:** Exocrine gland, Insect, Epithelial tube, Organogenesis, Cell polarity, Actin cytoskeleton, Apoptosis, Invagination

## Abstract

**Background:**

The hypopharyngeal gland of worker bees contributes to the production of the royal jelly fed to queens and larvae. The gland consists of thousands of two-cell units that are composed of a secretory cell and a duct cell and that are arranged in sets of about 12 around a long collecting duct.

**Results:**

By fluorescent staining, we have examined the morphogenesis of the hypopharyngeal gland during pupal life, from a saccule lined by a pseudostratified epithelium to the elaborate organ of adult worker bees. The hypopharyngeal gland develops as follows. (1) Cell proliferation occurs during the first day of pupal life in the hypopharyngeal gland primordium. (2) Subsequently, the epithelium becomes organized into rosette-like units of three cells. Two of these will become the secretory cell and the duct cell of the adult secretory units; the third cell contributes only temporarily to the development of the secretory units and is eliminated by apoptosis in the second half of pupal life. (3) The three-cell units of flask-shaped cells undergo complex changes in cell morphology. Thus, by mid-pupal stage, the gland is structurally similar to the adult hypopharyngeal gland. (4) Concomitantly, the prospective secretory cell attains its characteristic subcellular organization by the invagination of a small patch of apical membrane domain, its extension to a tube of about 100 μm in length (termed a canaliculus), and the expansion of the tube to a diameter of about 3 μm. (6) Finally, the canaliculus-associated F-actin system becomes reorganized into rings of bundled actin filaments that are positioned at regular distances along the membrane tube.

**Conclusions:**

The morphogenesis of the secretory units in the hypopharyngeal gland of the worker bee seems to be based on a developmental program that is conserved, with slight modification, among insects for the production of dermal glands. Elaboration of the secretory cell as a unicellular seamless epithelial tube occurs by invagination of the apical membrane, its extension likely by targeted exocytosis and its expansion, and finally the reorganisation of the membrane-associated F-actin system. Our work is fundamental for future studies of environmental effects on hypopharyngeal gland morphology and development.

**Electronic supplementary material:**

The online version of this article (doi:10.1186/s12983-017-0207-z) contains supplementary material, which is available to authorized users.

## Background

The European honey bee (*Apis mellifera*) forms highly organized colonies that function as a superorganism [17]. The majority of individuals in a bee colony, the sterile worker bees, support the queen, the drones, and the brood by undertaking various tasks in a temporal sequence [49]. During the first 2 weeks after their emergence, worker bees perform activities within the hive, i.e., cleaning cells, caring for the brood and the queen, ripening nectar, and constructing combs. As the worker bees age to 2-3 weeks, they assume extra-nidal tasks, in particular foraging for pollen, nectar, and water. These behavioral alterations accompany changes in transcriptional and translational activity, physiology, and morphology [9, 11, 18, 23, 30, 44, 46, 48, 49]. In particular, the hypopharyngeal gland in worker bees has a developmental cycle closely related to the division of labor. The paired hypopharyngeal gland is an exocrine gland specific to hymenopterans, is located in the front of the head capsule, and delivers its proteinaceous secretory product via a large collecting duct to the hypopharynx [7]. In nursing bees, this gland is voluminous, has a high secretory activity, and contributes to the production of royal jelly, which is fed to future queens and, to a lesser extent, to worker larvae [33]. As the worker bees start foraging, their hypopharyngeal glands decrease in size, secrete at a lower rate, and produce a different protein blend including enzymes involved in carbohydrate metabolism [9, 30, 44].

**Fig. 1:**
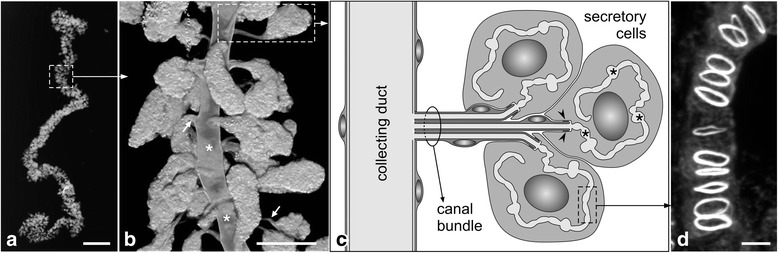
Hypopharyngeal gland in adult worker bee. **a** and **b** Macroscopic and microscopic views of hypopharyngeal glands. The gland consists of several hundred acini that are arranged around a collecting duct (*white asterisks*) and connected to the latter via a bundle of microcanals (*white arrows*). **c** Schematic presentation of the organization of an acinus. Each acinus is composed of several secretory cells that have their apical membrane involuted to form a long canaliculus (*black asterisks*). Each secretory cell is attached via a junctional complex (*arrowheads*) to a canal cell that forms a microcanal for the delivery of secretory products to the collecting duct. **d** The actin cytoskeleton along the membrane of the canaliculus. Maximum intensity projection of a confocal image stack through a secretory cell labeled with fluorophore-tagged phalloidin. Rings of actin filaments girdle the canaliculus at regular intervals. Faint staining for F-actin is associated with the inter-ring sections of the canaliculus. Bars, **a** 1 mm, **b** 100 μm, **d** 2.5 μm

The hypopharyngeal gland in worker bees has a characteristic morphology (Fig. 1). It is composed of thousands of two-cell units, a secretory cell and a duct cell [7, 22]. The secretory cell discharges its products into the canaliculus, a blind-ending membrane-bound tubule that meanders within the cell and that is covered on its lumenal side by a thin fenestrated cuticular lining termed the end apparatus [22, 35]. At the open end of the canaliculus, the secretory cell forms a tube-joint-like connection to the duct cell, a long thin ductule lined by a cuticular layer. Based on these morphological characteristics, hypopharyngeal glands thus belong to class III of the insect dermal glands [28, 29]. Groups of 6–20 two-cell units are clustered to form acini, with the duct cells extending in a bundle toward the collecting duct. In each hypopharyngeal gland, about 800 such acini are arranged around and along the 60-μm-wide collecting duct that delivers the secretion to the hypopharynx [7].

From a cell-biological perspective, the canaliculus of the secretory cells is peculiar. This structure has been suggested to represent the apical domain of the plasma membrane involuted into the cell [5]. Only recently, however, has molecular evidence been provided in support of this notion. Richter et al. [35] have demonstrated that phosphorylated (=activated) moesin, an apical membrane marker that links actin filaments to integral membrane proteins, is associated with and confined to the canalicular membrane. The cytoskeletal system affiliated to the canaliculus is also special. Rings of actin filaments encircle the membrane tubule at regular distances, whereas a sparse web of actin filaments is associated with the inter-ring portions of the canaliculus [21, 22, 35]. These actin rings are thought to provide a stabilizing framework to the canalicular membrane system during phases of high exocytic activity [22].

The anatomy and the cellular and subcellular organization of the hypopharyngeal glands in the adult worker bee have been studied extensively by use of various techniques, i.e., histology, electron microscopy, and fluorescence microscopy (e.g., [9, 16, 21–23, 31, 35]). In particular, several studies have addressed the structural changes that occur in hypopharyngeal gland as worker bees age and/or adopt other tasks [9, 23, 31, 35]. Of special interest is also the influence of pesticides on the morphology and physiology of adult hypopharyngeal glands and, thus, of the adverse side effects of these substances on honeybee vigor [16, 45]. In contrast, the organogenesis of the hypopharyngeal glands and the morphogenesis of the various gland cells have not been characterized as yet in detail. By use of histological techniques, Painter [32] has examined pupal gland cells, without noting the exact developmental stage, and has provided evidence for the transient presence of an additional cell type besides the secretory cell and duct cell during the morphogenesis of the gland units. Subsequently, da Cruz-Landim and Mello [8] have analyzed hypopharyngeal gland morphogenesis during pupal life by using histological techniques, but in the stingless bee *Melipona quadrifasciata anthidioides*. Since none of these studies has examined the morphogenesis of secretory cells at the subcellular level, the time and the manner in which the secretory cells form their distinctive apical membrane system, the canaliculus, remain mysterious. This topic is of genuine interest in view of the recent finding that insecticides impair brood development [45].

In the present study, we have attempted to track the origin of the secretory and duct cells during pupal hypopharyngeal gland development by using the DNA-binding dye DAPI and the F-actin-binding phalloidin to visualize nuclei and the cell outline, respectively. We confirm the transient existence of an additional cell type between the secretory cell and duct cell, and that this cell is lost at the mid-pupal stage by apoptosis. Moreover, we examine the development of the canalicular system and the establishment of the associated rings of actin filaments in secretory cells.

## Results

### Gross morphology of hypopharyngeal glands during pupal development

**Fig. 2:**
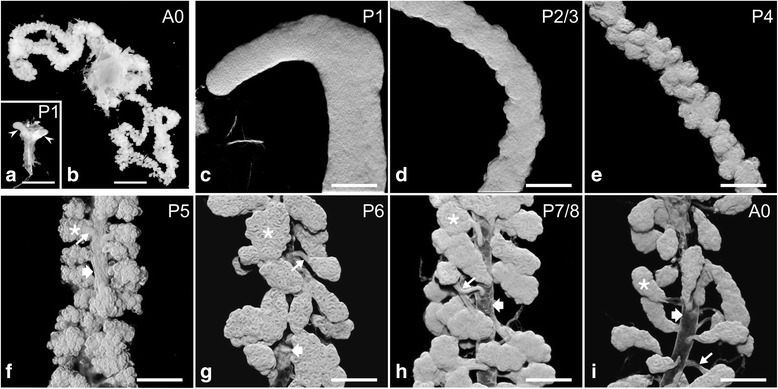
Hypopharyngeal gland morphogenesis. **a** and **b** Macroscopic images of hypopharyngeal gland primordia (arrowheads) at pupal stage P1 (**a**) and of hypopharyngeal glands in a newly emerged worker bee (**b**; A0). Note the size difference. **c**-**i** Microscopic views of hypopharyngeal glands during pupal development (P1-P7/P8) and of a newly emerged worker bee (A0). Confocal image stacks of phalloidin-labeled glands are presented in 3D shadow mode. Asterisks, acini; thin arrows, bundle of ductules; broad arrows, collecting duct. Bars, **a** and **b** 1 mm; **c**-**i** 100 μm

Morphogenetic events during hypopharyngeal gland development were studied by staining with fluorophore-conjugated phalloidin in conjunction with serial confocal sectioning and three-dimensional (3D) image reconstruction. Since F-actin is enriched on the plasma membrane [4], the morphology of entire hypopharyngeal glands can be depicted by using appropriate parameters for image acquisition and modes for 3D presentation (Fig. 2).

Pupae were staged from P1 to P9, equivalent to days of pupal development [12, 14]. At developmental stage P1, two saccule-like evaginations, representing hypopharyngeal gland primordia, extended from the ventral side of the pharynx (Fig. 2a and c). The saccules consisted of a transparent epithelium enclosing a large lumen, had a smooth outer surface, and measured about 0.5 mm in length and 0.2 mm in width. During subsequent days of pupal developmental up to stage P4, the hypopharyngeal gland primordia increased in length to about 5 mm, their width declined to about 0.1 mm, and their outer surface became undulating (Fig. 2d and e). By stage P5, a collecting duct of approximately 40 μm in width extended on the medial axis along the entire hypopharyngeal glands. Numerous cauliflower-like structures, representing future acini, were arranged around the duct, being linked to it by stalks that were approximately 20 μm long and 20 μm thick (Fig. 2f). Because of the large number of acini and their proximity to the duct and to each other, the collecting duct was almost completely masked from sight. By stage P6, the hypopharyngeal glands had adopted a gross morphology similar to that of adult glands (Fig. 2g-i), with numerous acini of ovoid shape linked via bundles of ductules of approximately 50 μm in length to the collecting duct that extended over the entire length of the gland.

### Mitotic events

**Fig. 3:**
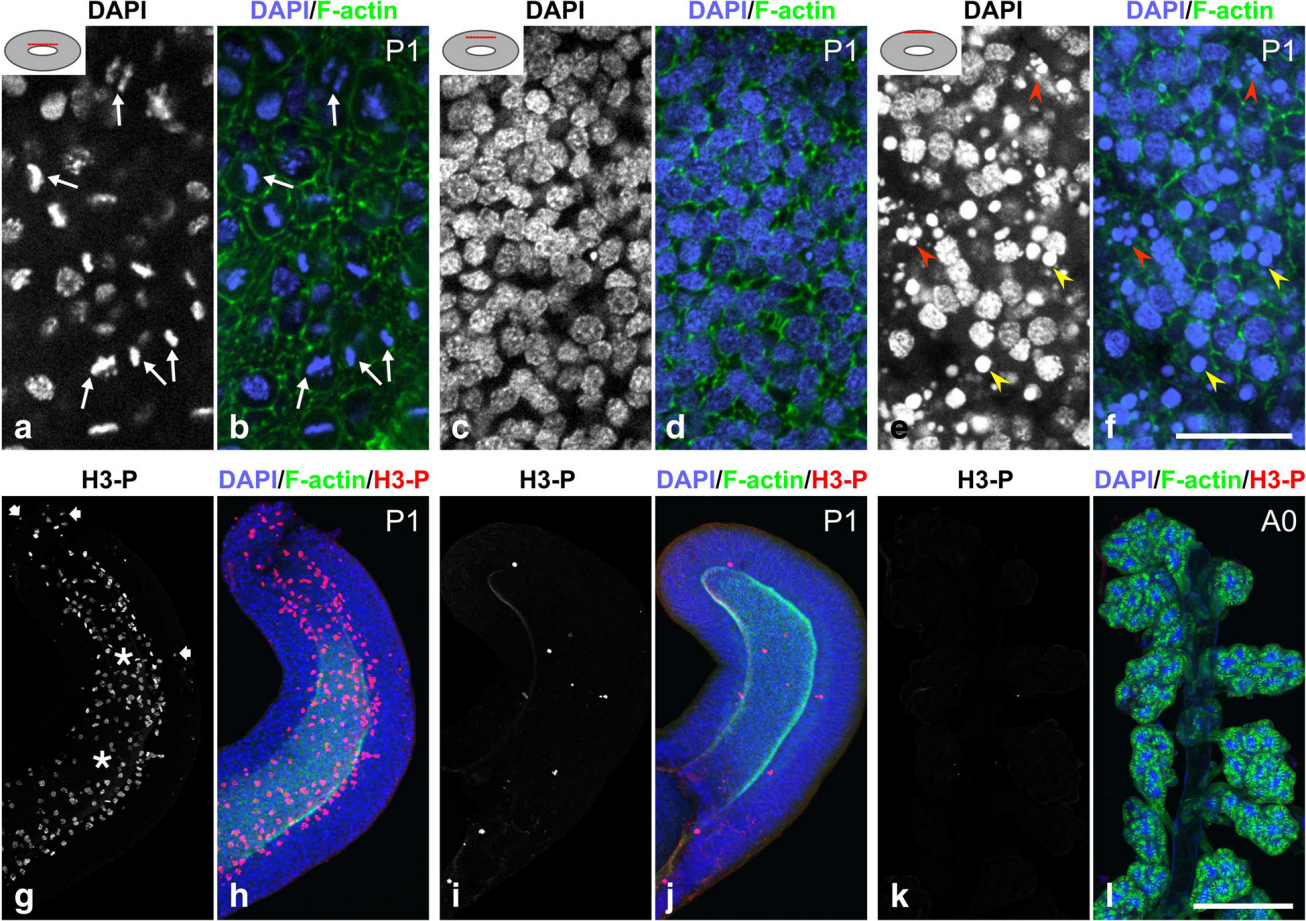
Mitotic cells in hypopharyngeal gland primordia at stage P1. **a**-**f** Optical sections at various levels (indicated by the *red* line in the inset) through a hypopharyngeal gland at stage P1 labeled with DAPI and fluorophore-tagged phalloidin (F-actin). **a** and **b** Few nuclei are located in the apical region of the epithelium, but most of these are in mitotic stages (*arrows*). **c** and **d** Mid-epithelial sections are crowded with interphase nuclei. **e** and **f** The basal region of the epithelium contains nuclei with condensed chromatin (*yellow* arrowheads), some of them seem fragmented (*red arrowheads*). **g** and **h** Hypopharyngeal gland of P1 pupa labeled with an antibody against phosphorylated histone 3 (H3-P), DAPI and, phalloidin (F-actin) and presented as maximum intensity projections. H3-P stained numerous nuclei close to the lumen (*asterisks*) and a few nuclei in the basal region of the epithelium (*arrows*). Bars, **a**-**f** 20 μm; **g** and **h** 100 μm


To identify mitotic events during hypopharyngeal gland development, entire glands were labeled with the DNA-binding dye 4′,6-diamidino-2-phenylindole (DAPI). At pupal stage P1, mitotic nuclei were detected in the apical portion of the epithelium, with the division plane in most but not all cases being oriented horizontally in the epithelial layer (Fig. 3a-f; Additional file 1). The middle and basal regions of the epithelium contained numerous interphase nuclei. In addition, the basal region had nuclei that contained condensed chromatin and that were sometimes fragmented, probably representing apoptotic cells. In order to validate the above results on the mitotic events in P1 gland primordia, organs were labeled with an antibody against histone H3 phosphorylated at Ser10 (H3-P; Fig. 3g and h). Anti-H3-P is known to be a reliable marker for mitosis in insect tissues [26, 27]. H3-P-positive nuclei were present in the apical region of the epithelium. Moreover, a few H3-P-positive nuclei were detected in the basal zone of the epithelium, suggesting that mitotic events also occurred in this region, although at low frequency. From stage P2/P3 on, no mitotic cells were detected by DAPI staining or anti-H3-P labeling. These results suggest that mitotic events are completed during the P1 phase.

Additional file 1: Animation of an image stack through a hypopharyngeal gland primordium, stained with phalloidin (green) and DAPI (blue). Whole-mount specimen; inter-plane distance, 0.34 μm; objective lens, Zeiss C-Apochromat 40x/1.2 W. See Fig. 3a-f for details. (AVI 10486 kb)


### Cellular morphology of developing hypopharyngeal glands

**Fig. 4:**
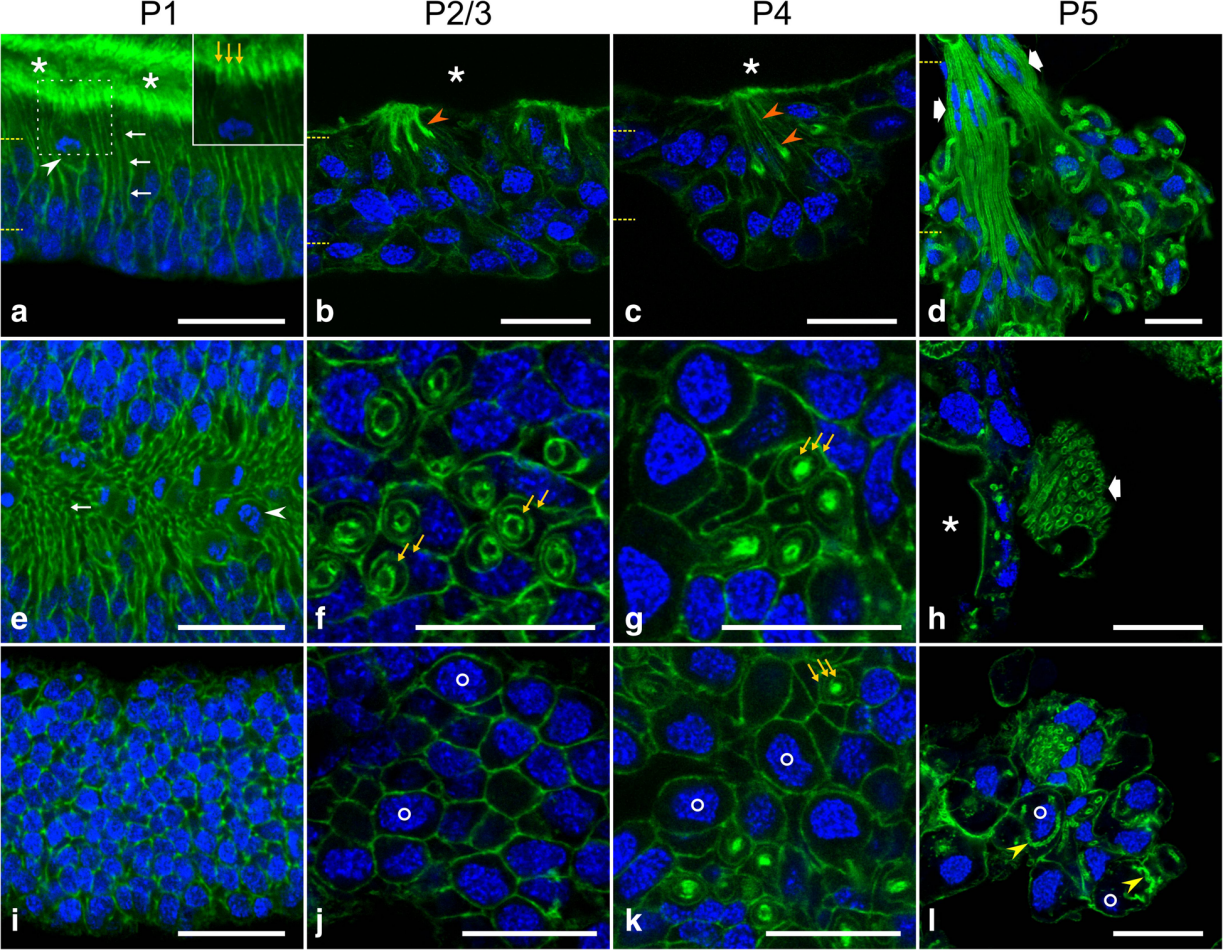
Differentiation of hypopharyngeal gland during the first half of pupal life. Glands were isolated, fixed, labeled with phalloidin (*green*) and DAPI (*blue*), and imaged by confocal serial sectioning. In the case of phalloidin images, gamma correction was set to 0.5 to visualize areas of faint staining. **a**-**d** Sagittal sections through the gland epithelium or acini at the developmental stages as indicated. Lumen of the gland primordium or the collecting duct is indicated by asterisks. **e**-**l** Horizontal optical sections through the epithelial layer or acini. Dashed lines in **a**-**d** indicate relative positions of section planes. At P1 (**a**,**e**,**i**), the hypopharyngeal gland primoridium is composed of a pseudostratified columnar epithelium with mitotic cells (arrowheads) in the apical region. Flask-like cells have their nucleus in the basal half and a narrow process (*white* arrows) extending toward the gland lumen. The area outlined by **a** dashed line is presented at higher magnification in the inset (*green*, no gamma correction). Intense phalloidin staining at the apicolateral side (*orange* arrows) indicates junctional complexes; the fainter staining between and above the apicolateral sides suggests the presence of microvilli. From P2-P4 on, F-actin-rich tubulous structures (red arrowheads) extend in bundles from the gland lumen basally and are wrapped by two to three concentric rings of cell processes (*yellow* arrowheads). Future secretory cells (circles) have a large nucleus in the basal region and are as yet devoid of canaliculus-like structures. At P5, the gland is organized into acini that are connected by bundles of ductules (broad arrows) to the collecting duct (asterisk). Future secretory cells (circles) contain an F-actin-rich tubulous structure, the future canaliculus (*yellow* arrowheads in l). Bars, 20 μm

In hypopharyngeal gland primordia at stage P1, the bounding epithelium was pseudostratified and about 40 μm thick (Fig. 4a, e and i). In addition to mitotic cells in the apical region, the epithelium consisted of flask-like interphase cells with their nuclei positioned at various levels in the mid and basal region of the epithelium. Interphase nuclei were oval and measured about 5 μm by 3 μm, with the long axis oriented in an apicobasal direction in the epithelial layer. A cellular process that was 1–2 μm thick and 10–20 μm long extended from the cell body to the luminal surface. Intense staining with phalloidin of the apicolateral sides of these processes indicated that F-actin occurred at adherens junctions (Fig. 4a inset). In addition, weaker staining over the entire apical surface of the cell processes suggested the presence of microvilli-like structures.

**Fig. 5:**
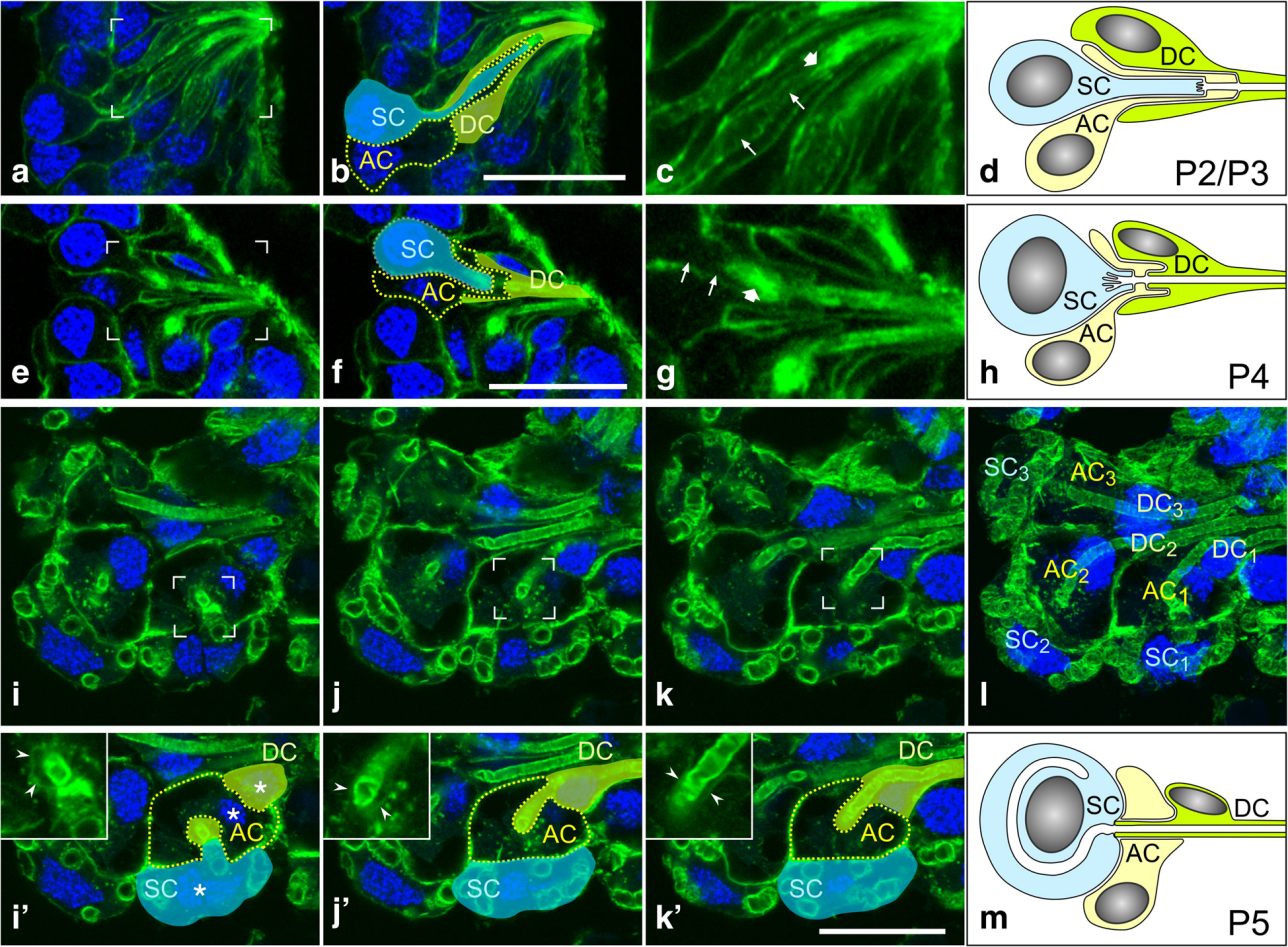
Gland units in pupal hypopharyngeals glands are composed of three cell types. Cryo-sections of hypopharyngeal glands were labeled with fluorophore-tagged phalloidin (*green*) and DAPI (*blue*) and imaged by confocal serial sectioning. Gamma correction was set to 0.5 for phalloidin images. Gland units were examined at developmental stages P2/P3 (**a**-**c**), P4 (**e**-**g**), and P5 (**i**-**l**). **d**, **h** and **m** Schematic presentations of the spatial arrangement of the secretory cell (SC), duct cell (DC), and accessory cell (AC) at developmental stages P2/P3, P4, and P5, respectively. **a**,**b** An optical plane visualizing a P2/P3 gland unit in longitudinal section, with the three cell types highlighted in (**b**). **c** The area outlined in **a** at higher magnification, showing the SC process (arrowheads) contacting (arrow) the distal end of the future ductule. **e** and **f** An optical plane visualizing a P4 gland unit in longitudinal section, with the three cell types indicated in (**f**). **g** The area outlined in **e** at higher magnification, showing the F-actin-rich terminus (arrow) of the short secretory cell process (arrowheads) that extends toward the future ductule. **i**-**k** Three image planes with an inter-plane distance of ~1.2 μm through an acinus at developmental stage P5. The indicated area is shown at higher magnification in the insets below. **i**’-**k**’ Lower regions of images i-k, with secretory cell (SC), accessory cell (AC), and duct cell (DC) outlined. Nuclei of the three cell types are indicated by asterisks. Insets in **i**’-**k**’ The distal part of the duct cell (arrowheads) is enclosed by the accessory cell. Arrowheads indicate the plasma membrane of the duct cell. **l** Maximum intensity projection of the entire image stack, representing a thickness of ~5 μm and showing three gland units. The presence of an acessory cell (AC_n_) is consistent for all secretory cell / duct cell assemblies. Bars, 20 μm


At developmental stage P2/3, the epithelium retained a uniform thickness of about 40 μm over its entire expanse (Fig. 4b). However, several cell types could be distinguished by their differences in morphology and position (Fig. 5a-d; Additional file 2). Of these, three different cells appeared to be organized into units, with each unit being characterized by a short F-actin-bounded tubule, probably representing the prototype of a ductule. Based on the layout of these three cells and their further morphogenesis, two of them were identified as the future duct cell and the secretory cell, respectively. The third cell type within the unit was interposed between the two above-mentioned cells and had no equivalence in the adult hypopharyngeal gland (subsequently, this cell is termed accessory cell). The body of the future secretory cell was located basally within the epithelium and contained a nucleus of about 6 μm in diameter (Figs. 4j and 5a,b). A cell process of 10–15 μm in length and 1–2 μm in width extended from the cell body in an apical direction to close off the distal end of the ductule. This secretory cell process was wrapped by two sheaths, the interior being formed by an accessory cell and the exterior by a future duct cell (Figs. 4f and 5a-d). The accessory cell had a nucleus of size and position similar to the future secretory cell. At the end of the secretory cell process, an extension of the accessory cell formed the distal portion of the ductule precursor. The nucleus of the prospective duct cell was smaller than the nuclei of the other cell types, being horizontally flattened and located in the mid-epithelial region (Fig. 5a and b). The future duct cell formed, in the basal portion of the three-cell unit, the outer sheath but reached with a narrow process above the end of the accessory cell process all the way to the luminal surface of the epithelium to build the proximal portion of the ductule precursor. Five to 15 of these three-cell units were arranged in clusters next to each other, with the ductule precursors extending in a radial fashion from the luminal surface basally for several micrometers (Fig. 4b). A few cells with a round nucleus in the apical portion of the epithelium were localized between the clusters and covered the remaining area of the luminal surface (Fig. 4b). We suggest that the clusters of the three-cell units represent future acini and their associated ductules, and that the intermediary cells will configure the collecting duct.

Additional file 2: Animation of an image stack through a hypopharyngeal gland at pupal stage P2/3, stained with phalloidin (green) and DAPI (blue). Cryosection; inter-plane distance, 0.37 μm; objective lens, Zeiss Plan-Apochromat 63x/1.4 Oil. See Fig. 5a-c for details. (AVI 514 kb)


At developmental stage P4, acini primorida bulged in a basal direction from the epithelial layer (Fig. 4c). Ductule precursors had increased in length to about 20 μm and were composed of a prospective duct cell almost over their entire length, except for a short segment lying next to the secretory cell and produced by an accessory cell (Fig. 5e-h; Additional file 3). The secretory cell process was shortened, retracted in a basal direction, and contained an onion-shaped F-actin-rich structure next to the distal end of the ductule. We consider that this structure corresponds to an array of microvilli and represents the origin for the development of the secretory cell canaliculus (see below).


Additional file 3: Animation of an image stack through a hypopharyngeal gland at pupal stage P4, stained with phalloidin (green) and DAPI (blue). Cryosection; inter-plane distance, 0.24 μm; objective lens, Zeiss Plan-Apochromat 63x/1.4 Oil. See Fig. 5e-g for details. (AVI 2590 kb)


**Fig. 6:**
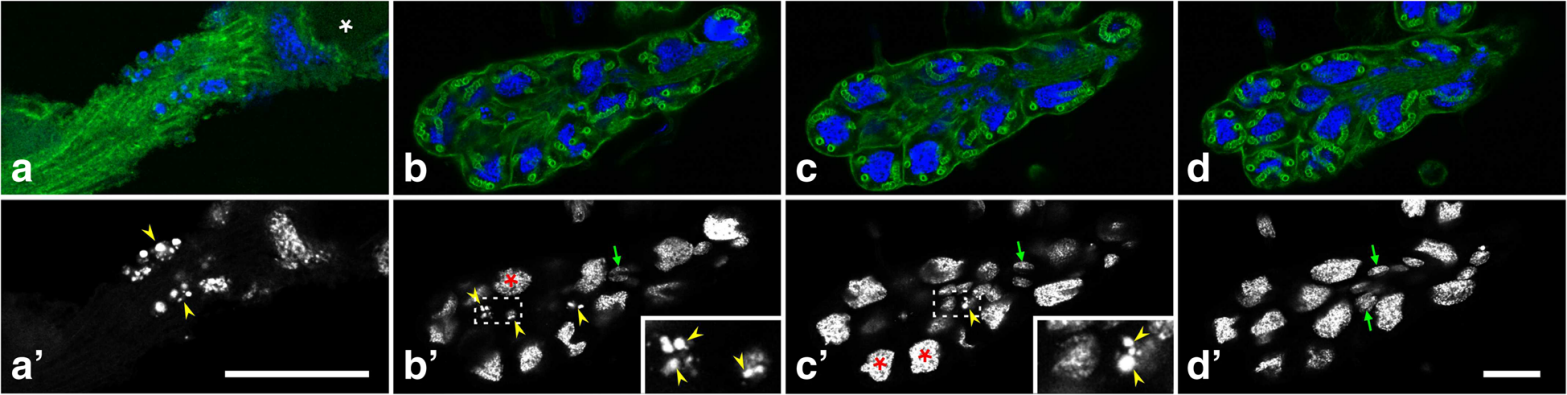
Apoptosis in hypopharyngeal gland acini at pupal stage P6. Glands were fixed, labeled with fluorophore-tagged phalloidin (*green*) and DAPI (*blue* in **a**-**d**, *white* in a’-d’), and imaged by confocal serial sectioning. **a** and **a**’ A bundle of ductules that connects the acinus (outside the field of view at the bottom left) with the collecting duct (*white* asterisk). **b**-**d** Three optical planes (inter-plane distance 3.8 μm) through an acinus. *Red* asterisks, secretory cell nuclei; *green* arrows, nuclei of duct cells or accessory cells; *yellow* arrowheads, nuclei with condensed and fragmented chromatin (shown at a higher magnification in insets). Bars, 25 μm

At developmental stage P5, the acini had moved basally out of the epithelial layer, remaining connected to it by short bundles of ductules (Fig. 4d). The remaining epithelial layer, the future collecting duct, was a monolayer of isoprismatic cells with an apical seam of F-actin, indicative of short microvilli (Fig. 4h). Rounded secretory cells with large nuclei were positioned on the periphery of the acini, whereas duct cell bodies with flattened nuclei were located in the interior of the acini or in the stalk between the ductules (Figs. 4d,l and 5i-l). The last-mentioned were formed over their entire length by duct cells, whereas accessory cells were restricted to a collar around the distal portion of the ductules, abutting the secretory cell (Fig. 5i-m; Additional file 4). Thus, except for the presence of accessory cells, P5 acini had adopted an organization similar to that of the hypopharyngeal glands of adult worker bees. Accessory cells seem to undergo apoptosis during pupal stages P6 to P8, since DAPI staining visualized fragmented nuclei in the interior of the acini and in the stalk (Fig. 6). Moreover, only secretory cells and duct cells were detected in gland units at later developmental stages.

### Differentiation of the canaliculus

Since the timing and manner of formation of the canaliculus of the secretory cells are unknown, we wished to analyze this morphogenetic process by the use of probes specific for this membrane domain. We have shown previously that anti-phosphorylated ERM (anti-pERM) and anti-phosphotyrosine selectively stain the canalicular system of adult secretory cells. Whereas anti-pERM outlines membrane segments between adjacent actin rings, anti-phosphotyrosine identifies dot-like structures that are associated with the canaliculus and that may represent microvillar tips [35]. Unfortunately, however, neither anti-pERM nor anti-phosphotyrosine stained any structures in prospective secretory cells of the pupal hypopharyngeal glands, suggesting that either these cells lack a canaliculus throughout pupal development, or that the expression of these proteins and/or their localization to the canalicular membrane occurs after the formation of the canalicular membrane system. Thus, we could only rely on the subcellular distribution of F-actin to probe the formation of the canalicular membrane, assuming that the F-actin assemblies in the interior of the secretory cells are associated with this membrane domain and/or its precursors.

**Fig. 7:**
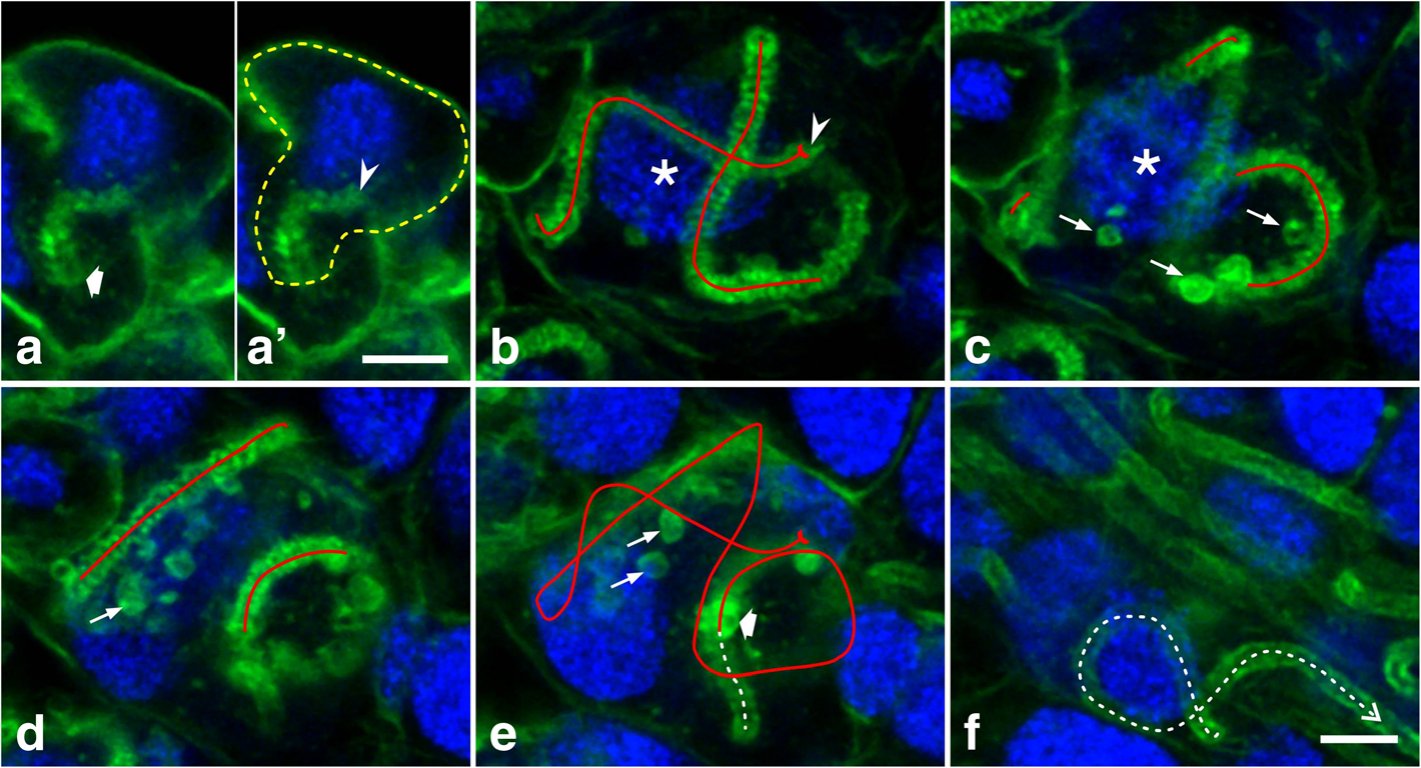
Canaliculi formation at pupal stage P5 proceeds from the ductule end. Entire glands were fixed, labeled with phalloidin (*green*) and DAPI (*blue*), and imaged by confocal serial sectioning. **a** and **a**’ In the secretory cell outlined by a dashed *yellow* line (**a**’), an array of F-actin defines a short tube that originates at the distal ductule end (broad arrow) and terminates blindly in the cytoplasm (arrowhead). Continuity between the F-actin tube and ductule was backtraced in confocal serial sections, although only one optical plane is shown here. **b**-**f** Five optical planes (inter-plane distance 1.15 μm) through a P5 acinus. A continuous blind-ending (arrowhead) tube is outlined by phalloidin staining and takes a convoluted path around the nucleus (asterisks) of the prospective secretory cell. Tube segments in each section plane are indicated by *red* lines and summarized in **e**. Spheroidal structures (thin arrows) close to the tube are also delineated by phalloidin staining. The F-actin tube is connected to the ductule (broad arrow); the latter is indicated by a dashed *white* line (**e**,**f**). Bars, 5 μm

**Fig. 8:**
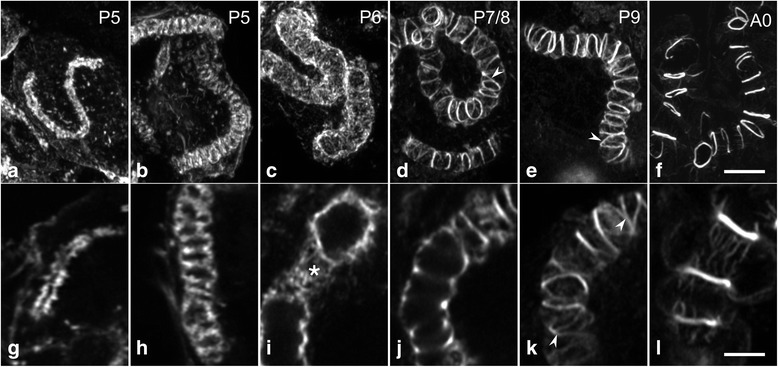
Morphogenesis of the canaliculus in secretory cells during the second half of pupal development. Cryosections through hypopharyngeal glands at various developmental stages were labeled with phalloidin and imaged by confocal serial sectioning. **a**-**f** Maximum intensity projections of image stacks. **g**-**l** Individual optical sections at a higher magnification. **a** and **g** At P5, the F-actin tube, representing F-actin associated with the developing canaliculus, is thin and short with a narrow lumen. **b** and **h** In developmentally more advanced P5 specimens, the F-actin tube has increased in length and diameter. As a result of periodic constrictions, the tube resembles a series of oblate spheroids on a string. **c** and **i** At P6, a dense web of F-actin (asterisk) forms an expanded tube of uniform diameter. **d**-**f** and **j**-**l** Between P7 and eclosion of the worker bees, F-actin in the tube becomes reorganized and concentrated into rings. Ring distance increases during developmental progression, whereas the number of interconnections (arrowheads) and the amount of F-actin in association with the inter-ring segments decreases. Bars, **a**-**f** 5 μm; **g**-**l** 2.5 μm


At developmental stages P2-P4, prospective secretory cells contained, at the contact site of their cell process with the ductule, an F-actin-rich structure (Fig. 5c, d, g and h). We interpret this structure as being an array of microvilli, as noted previously for developing secretory cells in female accessory glands in *Rhodnius prolixus* [24]. No other F-actin-rich assemblies were identified within the prospective secretory cells during these developmental stages, suggesting that the canaliculus had not yet formed. In P5 secretory cells, a single continuous tube-like F-actin structure extended from the basal terminal of the ductule into the secretory cell for various distances. In some specimens, the tube ended after a few micrometers (Fig. 7a; Additional file 5:). However, in the most extended version, the tube-like F-actin array had a length of about 100 μm and adopted a meandering path around the nucleus (Fig. 7b-f; Additional files 6 and 7), similar to the canaliculus in adult secretory cells [35]. We suggest that these F-actin tubes are associated with membrane on their inside, and tubes of increasing length represent sequential developmental stages of canaliculus formation. This length increase was accompanied by fine-structural changes (Fig. 8). Relatively short F-actin tubes had an external diameter of about 0.8 to 1.0 μm and an internal diameter of about 0.3 μm (Fig. 8a and g). Long F-actin arrays had a tube wall of uniform (apparent) thickness of 0.2 μm, although their outer diameter varied between ~1.2 and ~2.5 μm in a periodic manner. Hence, these long F-actin tubes had the appearance of a long series of conjoined oblate spheroids (Fig. 8b and h). Some individual F-actin spheres with a diameter of 1.0 to 2.5 μm were observed either in contact with or apart of the long F-actin tube (Fig. 7c-e). We consider that these structures represent material for the growth of the canaliculus.

Additional file 5: Animation of an image stack through a hypopharyngeal gland at pupal stage P5, stained with phalloidin (green) and DAPI (blue). Whole-mount specimen; inter-plane distance, 0.29 μm; objective lens, Zeiss C-Apochromat 40x/1.2 W. See Fig. 7a for details. (AVI 670 kb)



Additional file 6:Animation of an image stack through a hypopharyngeal gland at pupal stage P5, stained with phalloidin (green) and DAPI (blue). Whole-mount specimen; inter-plane distance, 0.29 μm; objective lens, Zeiss C-Apochromat 40x/1.2 W. See Fig. 4d for details. (AVI 8006 kb)



Additional file 7:Animation of an image stack through a hypopharyngeal gland at pupal stage P5, stained with phalloidin (green) and DAPI (blue). Whole-mount specimen; inter-plane distance, 0.29 μm; objective lens, Zeiss C-Apochromat 40x/1.2 W. This movie shows an area of the acinus presented in movie 6. See Fig. 7b-f for details. (AVI 411 kb)


At stage P6, the canaliculus was radially expanded to a diameter of 2.8 to 3.5 μm over its entire length, without any periodic constrictions. Canaliculus-associated F-actin was organized in a planar irregular web (Fig. 8c and i). Other than the canaliculus, no other F-actin assemblies were detected in the interior of the prospective secretory cells from this developmental stage on. By developmental stage P7/P8, canaliculus-associated F-actin was concentrated in closely spaced, frequently interconnected or fused rings, with a ring diameter of 2.5 to 3.0 μm (Fig. 8d and j). Areas between these F-actin assemblies were covered by a sparse matrix of actin filaments. During subsequent pupal development, the actin rings became more pronounced, the extent of the interconnections decreased, and the distance between the adjacent actin rings increased (Fig. 8e and k). In addition, the amount of F-actin associated with inter-ring portions seemed to decrease during the last few days of pupal development. Hence, canaliculi-associated actin rings were prominent and were regularly spaced with a few residual interconnections by the eclosion of the worker bees (Fig. 8f and l), as described previously [35].

## Discussion

**Fig. 9:**
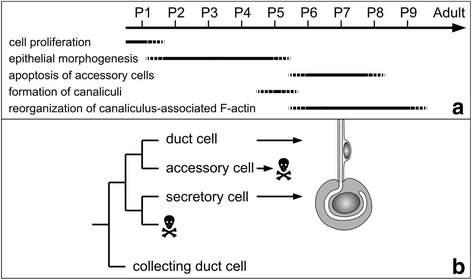
Schematic outline of key events during pupal development of hypopharyngeal gland. **a** Timeline for major developmental events. P1-P9, pupal stages, termed according to the literature [12, 14]. **b** Putative cell lineage in worker bee hypopharyngeal gland, based on the cell lineage of other insect dermal glands [38, 40] and modified by taking into account our findings

During the pupal development of worker bees, the hypopharyngeal gland primordium, which is a simple saccule enclosed by a pseudostratified epithelium, develops into an elaborate organ composed of hundreds of acini that are connected to and arranged around a collecting duct. Our results demonstrate that this developmental process can be subdivided into several key events (Fig. 9a): (1) During the first day of pupal life, at pupal stage P1, mitoses in the epithelial layer produce precursors for all the cells that compose the adult hypopharyngeal gland. (2) During pupal phase P2/P3, the epithelium becomes organized into rosettes of three cells, i.e., a prospective duct cell, an accessory cell, and a prospective secretory cell. Sets of five to 15 of these three-cell units become arranged in clusters within the epithelium. Eventually, such a patch of cells will differentiate into an acinus and its associated canal bundle, whereas cells between these clusters will produce the collecting duct epithelium. (3) By P5, morphogenesis of the three-cell units has produced a gland that is organized into hundreds of acini linked by canal bundles to a collecting duct, as in the adult hypopharyngeal gland. (4) Between pupal stages P6 to P8, the three-cell units are converted to two-cell units by the apoptotic elimination of the accessory cells. (5) During pupal stage P5, the canalicular system in the prospective secretory cells is formed by the invagination of a small apical domain, its extension to a tube of approximately 100 μm in length, and its expansion to a diameter of about 3 μm. (6) After the establishment of the canaliculus to its full extent, the membrane-associated F-actin system becomes reorganized and concentrated at regular distances along the canaliculus to form actin rings.

Hypopharyngeal glands, being specific to hymenopterans, are quite diverse in morphological aspects [7]. Whereas the two-cell unit of secretory cell and canal cell seems to be common, the modes in which these units are organized to hypopharyngeal glands vary between species. In particular, the units may be attached directly to the hypopharyngeal plate or deliver their secretory product via a more or less elaborate collecting duct to the hypopharynx. Moreover, two-cell units may be individually attached to the collecting duct, like in the stingless bee *Melipona quadrifasciata anthidioides*, or several two-cell units may be assembled to acini with bundled ductules, as in *Apis mellifera*. Unfortunately, hypopharyngeal gland development has been examined as yet only in *M. quadrifasciata anthidioides*, but by histological techniques [8]. Nevertheless, since all hypopharyngeal glands are built of two-cell units, it may be supposed that the developmental program leading to these units is also shared among hymenopterans.

### Development of secretory units

Dermal glands of insects exhibit an enormous diversity. On morphological criteria, they have been classified into three types, with class III being organized in basic units of secretory and support cells [28, 29]. The development of class III glands has been proposed to be based on a common mechanism. Each glandular unit is an isogenic group of four cells that are derived from one progenitor cell by sequential mitoses [29]. Initially, these cells are concentrically wrapped one around another with, first, the innermost cell termed either the ciliary or basal cell, then the future secretory cell, and finally, the two outermost duct-forming cells [34, 38]. Subsequent apoptosis leads to mature gland units composed of fewer than four cells; at minimum, just one cell remains as a secretory cell.

In the present study, we visualized mitotic cells in the hypopharyngeal gland primordium, but we did not track the developmental fate of daughter cells. Nevertheless, our data are congruent with the above model of class-III-gland development in insects. Since secretory units in the hypopharyngeal gland of adult worker bees are composed merely of two cells, namely a secretory cell and a duct cell, two cells seem to be lacking in the case of the four-cell isogenic group. One of the cells missing in the adult state is the accessory cell that resides between the future duct and secretory cells during the first half of pupal life. The wrapping of these three cells around each other, with the future duct cell being the outermost and the prospective secretory cell lying on the inside, is reminiscent of the situation in the developing mandibular glands of the death’s head cockroach *Blaberus craniifer* and tergal glands of the male German cockroach *Blattella germanica* [34, 38]. The presence of an accessory cell in pupal hypopharyngeal glands has previously been described [32]. Painter suggested that this cell, being transiently present, becomes “absorbed” by the secretory cell. Our data indicate, instead, an apoptotic fate for this cell after the formation and elaboration of the gland units. The other cell missing in the secretory units of the hypopharyngeal gland is the basal/ciliary cell that is located basal of the secretory cell in other insect glands. Conceivably, this cell is not produced in honeybee hypopharyngeal glands because of a change in cell lineage during gland evolution, as demonstrated for *Drosophila* spermathecae [40]. Alternatively, the basal cell may undergo apoptosis soon after its production and thus does not contribute to the further development of the gland units. In support of the second option are the numerous apoptotic nuclei in the basal region of the P1 epithelium, concurrently with mitotic events.

In summary, we suggest the following model of cell lineage for the secretory units in the worker bee hypopharyngeal gland (Fig. 9b): mitosis of a progenitor cell produces four cells; one of them, localized basally, undergoes apoptosis shortly after genesis, whereas another one, the accessory cell, contributes to secretory unit morphogenesis but suffers cell death during late pupal development. The remaining two cells form the gland units (see below).

### Development of the canaliculus

The two-cell secretory units of adult hypopharyngeal glands can be considered as epithelial tubes, with the tube lumen being circumscribed by the apical surface of the cells [35]. The canal cell forms a continuous conduit that opens into the collecting duct, whereas the adjoining secretory cell contains the blind ending of the tube. Electron-microscopic imaging has demonstrated that both cells are linked by an intercellular junctional complex, but that the cells do not form autocellular junctions [2, 5, 35]. These fine-structural details enable the secretory units of the hypopharyngeal gland to be classified as unicellular seamless tubes.

The morphogenesis of seamless epithelial tubes has been analyzed in three different model systems, i.e., the vertebrate vascular system, the tracheal system of *Drosophila*, and the excretory system of *Caenorhabditis elegans*. These studies have demonstrated three ways in which unicellular epithelial tubes can be created [36, 37, 41]. First, vesicular structures in the cytoplasm of the cell merge and form a lumen that extends over the entire length of the cell and fuses finally at its two ends with the plasma membrane. This mechanism, termed cell hollowing, is involved in the formation of vertebrate blood vessels and of the excretory canal cell in *C. elegans* [6, 19]. Second, a patch of apical membrane circumscribed by intercellular junctions enlarges by exocytosis and extends internally into the cell. Such apical invagination in combination with apically directed exocytosis has been detected in the case of the development of the tracheal terminal cells of *Drosophila* [13] and of the blood vessels of the zebrafish [15]. Finally, as in the case of the excretory duct cell of *C. elegans*, an epithelial cell wraps itself up with its apical surface towards the inside, forms an autocellular junction to close the tube, and subsequently removes the junction [39].

At P2/P3, the earliest time-point at which a tubular structure could be identified in the hypopharyngeal gland primordium, the short ductule was connected to the apical surface of the epithelium, was continuous, and was apparently formed by three cells arranged in a row, with the future duct cell and the accessory cell molding the tube and with the prospective secretory cell closing off the distal end of the tube. Unfortunately, we were unable to visualize the initial step of tube formation. However, since all cells in the pseudostratified epithelium reach to the luminal surface and hold an apical domain, cell wrapping or invagination may account for the creation of the ductule precursor. Discrimination between these possibilities requires the imaging of the junctional complexes. Since antibodies against *Drosophila* junctional proteins did not cross-react with their honeybee homologues [35], we are currently unable to characterize the initial process of ductule formation.

In the case of secretory cells and their canaliculus, our data are in agreement with an invagination and apically targeted exocytosis. At developmental interval P2 to P4, the prospective secretory cell contacts the distal end of the ductule with a small surface area that is enriched with F-actin. We suggest that the F-actin at this site reflects the presence of microvilli, as demonstrated in glands by electron microscopy of developing secretory cells in the female accessory of *Rhodnius prolixus* [24]. During the P5 stage, a continuous tube grows from this site inwards into the future secretory cell to reach a final length of about 100 μm. Spheroidal F-actin structures in the vicinity of the developing tube are indicative of membrane material for tube extension.

In various insect dermal glands, yet another, completely different mechanism has been reported for the formation of the canaliculus of secretory cells [3, 34, 38]. A basal cell, located basally to the future secretory cell, extends a ciliary process that pierces the future secretory and duct cells to form a mold for the canaliculus and the duct. Subsequently, the ciliary process retracts and the basal cell degenerates. Although we did not attempt to localize cilia, such a mechanism can be rejected in the case of hypopharyngeal gland morphogenesis since basal cells are absent, at least during the period of canaliculus formation. Similarly, ductule morphogenesis in the female accessory glands in *Rhodnius prolixus* and in *Drosophila* spermathecae has been reported to occur without the contribution of a ciliary process [24, 40].

After the generation of the canaliculus in full length in the secretory cell, the tube becomes elaborated. Accordion-like folding of the tube wall at this developmental stage indicates an increase in surface area, apparently as a stockpile for later expansion. The presence of F-actin-bounded vesicular structures in the cytoplasm alongside the canaliculus during this developmental stage indicates that the surface increase is fed by the lateral fusion of vesicles. Likewise, new membrane material is added by way of vesicles along the tube of the apical membrane in the terminal tracheal cells of *Drosophila* [13]. Subsequently, the canaliculus expands to a uniform diameter of about 3 μm. This process may be driven by ion and water transport into the canaliculus, like the aquaporin-dependent increase in lumen size in the excretory canal cell of *C. elegans* [20]. Alternatively or concomitantly, the canaliculus might become inflated and stabilized by the deposition of chitin in the lumen [10, 37, 43].

### Differentiation of the F-actin system associated with the canalicular membrane

A network of F-actin is generally associated with the luminal membrane of epithelial tubes [13, 37]. This feature enabled the imaging of the developing canaliculus in the hypopharyngeal gland secretory cells, despite antibodies against marker proteins for the apical membrane not working in the present study. We have demonstrated that the F-actin system that is attached to the canalicular membrane is reorganized during the second half of pupal life. At developmental stage P6, when the canalicular system is formed to its full extent, a web of actin filaments with a seemingly random orientation surrounds the canalicular tube. F-actin then becomes gradually concentrated in rings, with the amount of interconnections decreasing and the inter-ring distance increasing. This change in actin cytoskeletal organization probably is concomitant or is attributable to a switch in the actin-binding proteins (ABPs) associated with the actin filaments on the canalicular membrane. Whereas cross-linking ABPs such as spectrin produce orthogonal arrays of actin filaments, bundling ABPs such as the Kelch protein of *Drosophila* can produce tight bundles of parallel actin filaments [47]. In agreement with this hypothesis is the finding that spectrin is not detectable with canaliculus-associated F-actin in adult secretory cells [35]. Moreover, the formation of actin rings in the ovarian ring canals of *Drosophila* depends on Kelch [42]. The expression of hDKIR, a human homologue of the *Drosophila* Kelch protein, produces ring-like actin structures in cultured mammalian cells [25].

Actin rings seem to be characteristic of secretory cells in hymenopteran hypopharyngeal glands. However, species-specific differences occur with respect to the diameter, the level of interconnections, and the distance between the actin rings [1, 22]. In particular, in the stingless bee *Tetragonula carbonaria*, actin rings on the canaliculus have frequent connections and are often not closed [22], thus resembling the P7/P8 intermediate stage in worker bees. We suggest that the differences in the relative expression of cross-linking and bundling ABPs account for these differences in actin ring organization between species.

## Conclusions

We have described the various steps of hypopharyngeal gland development from the pupal primordium to the intricate organ that adult worker bees possess at emergence. The gland develops as follows: cell proliferation in a pseudostratified epithelium, formation and morphogenesis of three-cell units within the epithelial layer, removal of accessory cells from the three cell units to obtain the final units of a duct cell and a secretory cell, elaboration of the canaliculus in the latter cell by invagination, extension and expansion of apical membrane, and finally reorganization of the canaliculus-associated actin cytoskeleton to form distinctive actin rings. Based on these findings, the effects of environmental factors, such as insecticides, on gland development can be explored. Moreover, since species-specific differences in the organization of the canaliculus-associated F-actin system have been reported, an analysis of hypopharyngeal gland development in other hymenopteran species might be informative.

## Methods

### Animals and preparation

Pupae of worker bees (*Apis mellifera*) were taken from combs with sealed broods and were kept in a humidified incubator at 34 °C. Pupae were staged from P1 to P9 by using morphological criteria, i.e., pigmentation of the eyes, bodies, and legs [12, 14]. Pupal stages P2 and P3 and stages P7 and P8 could not be discriminated unambiguously; hence, we pooled these stages into P2/P3 and P7/P8, respectively. Newly emerged worker bees (stage A0) were collected off the comb just after emergence. Animals were decapitated, the head capsule was opened on the frontal side with a microscalpel, and the hypopharyngeal glands were removed in Ringer solution (270 mM NaCl, 3.2 mM KCl, 1.2 mM CaCl_2_, 10 mM MgCl_2_, 10 mM morpholinopropansulfonic acid, pH 7.3) and immediately transferred to fixative (3% paraformaldehyde, 1 mM dithiobis(succinimidyl proprionate), 0.1 M phosphate buffer, pH 7.0).

### Antibodies

The following antibodies were used: monoclonal rabbit antibody against phosphorylated ezrin/radixin/moesin (pERM; product # 3149; Cell Signaling, Danvers, MA, USA), monoclonal mouse antibody clone PY99 against phosphotyrosine (pY; Santa Cruz Biotechnology Inc., Santa Cruz, CA, USA), and polyclonal rabbit antibody against histone H3 phosphorylated at Ser10 (H3-P; product # 06–570; Merck Millipore, Billerica, MA, USA). Cross-reactivity of anti-pERM with honeybee moesin has been demonstrated previously [35].

### Fluorescence staining and imaging

After fixation for 1 h at room temperature, specimens were washed, cryofixed in melting isopentane (ca. -150 °C), cryosectioned at a thickness of about 10 μm, and stained with antibodies, 4′,6-diamidino-2-phenylindole (DAPI), and AlexaFluor 488 phalloidin (Life Technologies GmbH; Darmstadt, Germany) or CF488A phalloidin (Biotium Inc., Hayward, CA, USA) as described in detail previously [50]. To label entire glands, fixed glands were (1) washed 3 × 10 min in phosphate-buffered saline (PBS), (2) permeabilized with 0.01% Tween20 in PBS for 10 min, (3) treated with 50 mM NH_4_Cl in PBS for 10 min, and (4) washed 1 × 10 min in PBS. After (5) treatment with blocking solution (1% normal goat serum, 0.8% bovine serum albumine, 0.5% Triton X-100 in PBS) for 15 min, specimens were (6) incubated over-night at 4 °C with anti-H3-P diluted in blocking solution, (7) washed 3 × 15 min in PBS, (8) incubated for 3 h at room temperature with Cy3-conjugated goat anti-rabbit IgG, fluorophore-tagged phalloidin, and DAPI in PBS, (9) washed again 3 × 15 min in PBS, and (10) embedded in Mowiol 4–88 mounting medium supplemented with 2% propyl gallate as an antifade reagent. In the case of the labeling of entire glands with phalloidin and DAPI only, steps 2–7 were omitted, and the specimens were incubated for 3 h or over-night with fluorophore-tagged phalloidin and DAPI, diluted in blocking solution. Fluorescence images were recorded with LSM 510, LSM 710, or LSM 880-Airyscan confocal microscopes and processed (3D presentation, gamma correction as noted in figure legends) with ZEN software (Carl Zeiss Microscopy GmbH, Jena, Germany).

## Additional files

Additional file 4: Animation of an image stack through a hypopharyngeal gland at pupal stage P5, stained with phalloidin (green) and DAPI (blue). Cryosection; inter-plane distance, 0.23 μm; objective lens, Zeiss Plan-Apochromat 63x/1.4 Oil. See Fig. 5i-l for details. (AVI 1144 kb)

## Abbreviations

ABPs: Actin-binding proteins
DAPI: 4′,6-diamidino-2-phenylindole
H3-P: Histone H3 phosphorylated at Ser10
PBS: Phosphate-buffered saline
pERM: Phosphorylated ezrin/radixin/moesin
